# Osmolality (mosmol/kg H_2_O) versus osmolarity (mosmol/L): applied physiology to improve patient safety

**DOI:** 10.1186/s40001-025-03652-7

**Published:** 2025-12-07

**Authors:** Rolf Zander, Thomas Ziegenfuß, Robert Sümpelmann

**Affiliations:** 1 Physioklin, formerly Institute of Physiology and Pathophysiology, University Medical Center, Mainz, Germany; 2 Department of Anesthesiology and Intensive Care Medicine, St. Josef Krankenhaus GmbH, Moers, Germany; 3 Clinic of Anaesthesiology, Hannover Medical School, Carl-Neuberg-Str.1, 30625 Hannover, Germany

**Keywords:** Osmolality, Osmolarity, Tonicity, Infusion solutions, Hyponatremia

## Abstract

Severe osmotic pressure imbalances may be associated with substantial morbidity and mortality. As a consequence, osmolality should be a routine parameter in clinical medicine, but actual clinical practice often looks different, and confusion or inaccurate interpretations are common. This paper therefore reviews the physiological background of osmolality to achieve a better understanding and to prevent complications in hospitalized patients. The use of osmolality (mosmol/kg H_2_O) instead of osmolarity (mosmol/L) is recommended for clinicians to avoid potentially misleading results when comparing solutions with different water contents. The manufacturers of infusion solutions should present the calculated actual osmolality instead of the theoretical osmolarity in the Summaries of Product Characteristics to provide a reliable foundation for clinical practitioners. To avoid unwanted water shifts, intravenous fluids for replacement of extracellular fluid or plasma should be isotonic with a calculated actual (in vivo) osmolality of 280 to 300 mosmol/kg H_2_O. Hypoosmolality is almost always caused by low sodium concentrations, and recent evidence suggests that slower rates of correction are associated with increased mortality compared to rapid correction.

**Supplementary Information:**

The online version contains supplementary material available at 10.1186/s40001-025-03652-7.

## Introduction

The osmotic pressure of body fluids is regulated precisely within narrow ranges (SD < 5.1 mosmol/kg H_2_O) by the human body. This is important, because small changes in osmotic pressure may lead to significant shifts of water across the cell membranes if body fluids are not in osmotic equilibrium. As a consequence, osmolality should be a routine parameter in clinical medicine, because severe osmotic pressure imbalances may be associated with substantial morbidity and mortality (see below). Unfortunately, actual clinical practice often looks different, and confusion or inaccurate interpretations are common (see Appendix with examples of publications with wrong or inconsistent units for or definitions of osmolarity, osmolality or tonicity). Therefore, this paper reviews the parameters osmolality (mosmol/kg H_2_O) and osmolarity (mosmol/L) to achieve a better understanding of the underlying physiology and of the diagnostic value in clinical medicine.

## Physiology of osmotic equilibrium

Different body compartments are in osmotic equilibrium if the number of osmotically active particles (osmoles) within the available water space is balanced. For example, freely permeable glucose is in osmotic equilibrium between erythrocytes (water content 71%) and plasma (water content 94%) if the concentrations in the different water spaces are similar. The osmolar concentration of a solution is called osmolality if the concentration is expressed as osmoles per kilogram of water (mosmol/kg H_2_O) and osmolarity if it is expressed as osmoles per liter of solution (mosmol/L). From a physiological point of view, the reference to kilogram of water (osmolality; mosmol/kg H_2_O) is essential because the reference to liter of solution (osmolarity; mosmol/L) may lead to confusing results when comparing solutions with different water contents. As examples, a glucose concentration of 5 mmol/L in plasma (water content 94%) would be in osmotic equilibrium with 2 mmol/L in erythrocytes (water content 71%), or a lactate concentration of 6 mmol/L in whole blood would be in osmotic equilibrium with 14 mmol/L in plasma (partition coefficient between eythrocytes and plasma 0.423) [1]. In contrast, previous experimental studies have shown that the values of osmolality (mosmol/kg H_2_O) of different body solutions including erythrocytes were almost the same [2].

## Determination of osmolality and osmolarity

The theoretical osmolarity of plasma can be calculated by adding up all osmotically active substances (binding to proteins excluded) relative to 1 L of plasma [3].

1 $$\begin{gathered} {\text{Theoretical osmolarity }}\left( {{\text{plasma}};{\text{ mosmol}}/{\text{L}}} \right) \, = {\text{ Na}}^{ + } + {\text{ K}}^{ + } + {\text{ Ca}}^{{{2} + }} + {\text{ Mg}}^{{{2} + }} + {\text{ Cl}}^{ - } + {\text{ HCO}}_{{3}}^{ - } + \, \hfill \\ {\text{lactate }} + {\text{ phosphate }} + {\text{ sulfate }} + {\text{ organic acids }} + {\text{ proteinate }} + {\text{ glucose }} + {\text{ urea }}\left( {{\text{all in mmol}}/{\text{L}}} \right) \hfill \\ \end{gathered}$$

As electrolytes, especially sodium and chloride, are only partially osmotically active (92.6% for sodium and chloride), the actual (real) osmolality of plasma can be calculated as a product of the theoretical osmolarity (see above) and an osmotic coefficient (0.926) divided by the water content (94%). [3]

2 $${\text{Actual osmolality }}\left( {{\text{plasma}};{\text{ mosmol}}/{\text{kg H}}_{{2}} {\text{O}}} \right) \, = \, \frac{{{\text{theoretical osmolarity }} \times {\text{ osmotic coefficient }}\left( {0.{926}} \right)}}{{{\text{water content }}\left( {0.{94}} \right)}}$$

For example, the sum of 142 (Na^+^), 4.5 (K^+^), 1.3 (Ca^2+^), 0.7 (Mg^2+^), 103 (Cl^−^), 24 (HCO_3_^−^), 1.5 (lactate), 1.0 (phosphate), 0.5 (sulfate), 1.5 (organic acids), 1.0 (proteinate), 5 (glucose) and 5 (urea; all in mmol/L) results in a theoretical osmolarity of 291 mosmol/L [3, 4]. The theoretical osmolarity of 291 mosmol/L multiplied by an osmotic coefficient of 0.926 and divided by the water content of 0.94 results in an actual (real) osmolality of 287 mosmol/kg H_2_O [5].

Surprisingly, the normal value of plasma osmolality of 288 mosmol/kg H_2_O measured by freezing point depression is – purely by coincidence – nearly identical for both the calculated plasma osmolality (287 mosmol/kg H_2_O) and the calculated osmolarity (291 mosmol/L). This coincidence is presumably responsible for some of the confusion in medical literature (see Appendix with examples of publications with wrong or inconsistent units for or definitions of osmolarity, osmolality or tonicity). Notably, calculating the theoretical osmolarity (mosmol/L) and actual (real) osmolality (mosmol/kg H_2_O) is also possible with infusion solutions. For examples, the actual (real) osmolality of saline 0.9% is the product of its theoretical osmolarity (308 mosmol/L = sodium 154 mmol/L + chloride 154 mmol/L) and its osmotic coefficient (0.926) divided by the water content (0.997), resulting in a value of 286 mosmol/kg H_2_O (= isotonic); the actual (real) osmolality of lactated Ringer’s solution is the product of its theoretical osmolarity (276 mosmol/l) and its osmotic coefficient (0.926) divided by the water content (0.997), resulting in a value of 256 mosmol/kg H_2_O (= hypotonic; Table 1).

**Table 1:** Osmolarity and osmolality of plasma and various infusion solutions

		Unit	Plasma	NaCl^1^	RL^2^	BS^3^	Glc 5%
Cations	Na +	mmol/L	142	154	130	145	–
	K^+^		4.5	–	5	4	–
	Ca^2+^		2.5	–	1	2.5	–
	Mg^2+^		1.25	–	1	1	–
Anions	Cl^–^	mmol/L	103	154	112	127	–
	HCO_3_^–^		24	–	–	–	–
	Acetate		–	–	–	24	–
	Lactate		1.5	–	27	–	–
	Malate		–	–	–	5	–
Glucose		mmol/L	5	–	–	–	278
Theoretical osmolarity		mOsmol/L	291^4^	308^5^	276^5^	309^5^	278^6^
In-vivo osmolality		mOsmol/kg H_2_O	288^7^	286^8^	256^8^	287^8^	0^9^

^1^Saline 0.9%

^2^Ringer’s lactate

^3^Balanced isotonic electrolyte solution

^4^including glucose, urea and organic acids

^5^Σ (cations + anions)

^6^Σ (glucose)

^7^measured by freezing point depression

^8^ = osmolarity × osmotic coefficient 0.926/water content 0.997

^9^after glucose metabolization

## Normal values of measured plasma osmolality

Importantly, freezing point depression (FPD) is currently the only method for the measurement of osmolality using two reference points: distilled water (0 mosmol/kg H_2_O) with an FPD of 0 °C and 1 osmol/kg mannitol solution (1,000 mosmol/kg H_2_O) with an FPD of -1.86 °C. Previous studies have shown that the mean value of the actual plasma osmolality measured by freezing point depression is about 288 mosmol/kg H_2_O with a low standard deviation between 0.9 and 5.1 mosmol/kg H_2_O (Table 2) [6–15]. Normal osmolality is independent of age, and similar in children and adults [16]. Interestingly, the results of measurements of osmolality in whole blood and plasma were comparable [17].

**Table 2:** Results of different studies measuring normal values of plasma osmolality (mosmol/kg H_2_O) in adults [6–14] and children [15] (no claim of completeness)

Osmolality (± SD)	n	References	Years
288 ± 5.0	181	[6-8]	1957–1973
285- 295		[9]	1968
290 ± 4.7		[10]	1972
286 ± 0.9		[11]	1974
289		[12]	1975
288 ± 4.9	100	[13]	1987
288.8 ± 3.4	41	[14]	2013
285.8 ± 5.1	280	[15]	2022

## Formulas for the calculation of plasma osmolality in clinical practice

A variety of formulas for calculating plasma osmolality in clinical practice have been published, most of them relying on sodium, urea and glucose [18]. A novel formula developed by Zander has recently been presented, which also takes into account the effects of potassium, chloride, lactate and bicarbonate on osmolality [4].

3 $${\text{Osmolality }}({\text{plasma}};{\text{ mosmol}}/{\text{kg H}}_{{2}} {\text{O}}) \, = \, \left( {{\text{Na}}^{ + } + {\text{ K}}^{ + } + {\text{ Cl}}^{ - } + {\text{ lactate }} + {\text{ glucose }} + {\text{ urea }} + {\text{ HCO}}_{{3}}^{ - } \left( {{\text{actual}};{\text{ all in mmol}}/{\text{L}}} \right) \, + { 6}.{5}} \right) \, \times \, 0.{985}$$

In an observational study comparing 36 different formulas, this formula showed the closest concordance with the measured osmolality, and facilitates a precise and rapid diagnosis based on blood gas analysis in clinical routine [14].

## Osmolar gap

The difference between the calculated and the measured plasma osmolality is defined as the osmolar gap [13]. A difference of greater than 10 is considered to be an elevated osmolar gap. Possible causes are the presence of 'pseudo'-hyponatremia, where plasma water levels deviate from normal values due to hyperlipidemia or hyperproteinemia, or the presence of unmeasured osmotically active compounds such as ethanol, methanol, isopropyl alcohol, ethylene glycol, paraldehyde, ether, trichloroethane, acetone, mannitol, glycerol, sorbitol, glycine, fructose or other unidentified osmotic substances, e.g., in chronic renal failure or shock. Importantly, the calculation of the osmolar gap may be useful in the differential diagnosis of patients presenting in emergency departments with possible drug or substance overdose or in hospitalized patients with coma [19].

## Tonicity

Tonicity is a dimensionless comparative term (for which there is no unit) that predicts changes in cell volume at equilibrium after exposure of the cells to a solution. A hypertonic solution causes water to leave the cells, while a hypotonic solution causes the cells to swell. Net water movement stops when the concentrations of osmotically active solutes in the cells and the solution are equal (= isotonic) [20]. To avoid unwanted water shifts, intravenous fluids for replacement of extracellular fluid or plasma should be isotonic to plasma (288 mosmol/kg H_2_O) with a calculated actual (in vivo) osmolality of 280 to 300 mosmol/kg H_2_O.

Interestingly, erythrocytes can be used as biological osmometers to evaluate changes in tonicity. After adding erythrocytes to a hypotonic or hypertonic solution, the change in cell volume will lead to a change of the measureables mean corpuscular volume (MCV), mean corpuscular hemoglobin concentration (MCHC) and the relation between hemoglobin concentration and hematocrit (cHb/Hkt). For example, a decrease in tonicity will lead to an increase in MCV when the erythrocytes are swelling and to a decrease in MCHC when hemoglobin is diluted in the swollen erythrocytes. An increase in tonicity has the opposite effects. In accordance, a previous laboratory study by Zander et al. (unpublished results; n = 25) showed, that the introduction of human erythrocytes into saline 0.9% (osmolality 286 mosmol/kg H_2_O) results in a measurable decrease in MCHC of 1 ± 0.9% when compared to plasma (osmolality 288 mosmol/kg H_2_O;). This simple experiment shows, that even small changes in osmolality have an impact on cell volume, that the use of the actual osmolality (mosmol/kg H_2_O) is suitable for describing tonicity when compared to the normal value of osmolality of 288 mosmol/kg H_2_O, and that changes in the hemogram can be used to evaluate changes in tonicity in clinical practice.

## Osmolality of infusion solutions

Unfortunately, most manufacturers of infusion solutions provide only the theoretical osmolarity (e.g., sum of anions and cations; mosmol/L) and not the calculated actual osmolality (mosmol/kg H_2_O). Therefore, it is difficult for clinical practitioners to evaluate the tonicity of a solution unless they are familiar with the complex relationship between theoretical osmolarity and actual osmolality. To maintain stable plasma osmolality, the theoretical osmolarity of an infusion solution should be close to 308 mosmol/L (as saline 0.9%) and the actual osmolality should be close to 288 mosmol/kg H_2_O (as plasma). The formula to calculate the actual osmolality of an infusion solution is presented above. To provide a reliable foundation for clinical practitioners, it is recommended that manufacturers should present the calculated actual osmolality instead of the theoretical osmolarity in the Summaries of Product Characteristics for their infusion solutions. Infusion solutions with an osmolality of 280 to 300 mosmol/kg H_2_O should be evaluated as isotonic, those with less than 280 mosmol/kg H_2_O as hypotonic, and those with more than 300 mosmol/kg H_2_O as hypertonic [21]. As examples, saline 0.9% is isotonic with an osmolality of 286 mosmol/kg H_2_O, lactated Ringer’s (Hartmann’s) solution is (very) hypotonic with an osmolality of 256 mosmol/kg H_2_O and glucose 5% is (extremely) hypotonic with an in vivo osmolality of 0 mosmol/kg H_2_O (= pure water; Table 1). In general, medical companies and manufacturers should provide physiologically composed isotonic balanced infusion solutions and include clear and detailed guidance for their safe and effective use. These relatively simple steps can be achieved without increasing costs and will have a substantial clinical benefit in reducing morbidity and mortality (see below) [22, 23].

## Osmolality in vitro and in vivo

Infusion solutions containing metabolizable osmotically active substances (e.g., glucose, acetate or lactate) may have different osmotic effects before infusion in the container (in vitro) and after administration of the infusion to the patient (in vivo). For example, an electrolyte-free 5% glucose solution contains 278 mmol/L glucose and has a theoretical osmolarity of 278 mosmol/L. The actual in vitro osmolality of this 5% glucose solution is the product of its theoretical osmolarity (278 mosmol/L) and its osmotic coefficient (1.013; different from saline 0.9%) divided by the water content (0.97), resulting in a value of 290 mosmol/kg H_2_O (= isotonic) [5]. After infusion, the glucose is metabolized rapidly inside the tissue cells and is not osmotically active thereafter, whereas the water remains in the body. In consequence, the in vivo osmolality of an electrolyte-free 5% glucose solution after metabolization is 0 mosmol/kg H_2_O (= pure water; Table 1). In infusion solutions containing acetate or lactate as bicarbonate precursors, the in vivo osmolality will be unchanged because the intracellular acetate or lactate metabolism results in an equimolar release of osmotically active bicarbonate. Infusion solutions containing malate or citrate have a higher in vivo osmolality, because the metabolization of 1 mmol malate leads to the release of 2 mmol bicarbonate and the metabolization of 1 mmol citrate leads to the release of 3 mmol bicarbonate. To provide a reliable foundation for clinical practitioners, it is suggested that the manufacturers should also present the calculated in vivo osmolality instead of the theoretical osmolarity in the Summaries of Product Characteristics for infusion solutions containing metabolizable substances in order to reduce the risk of dangerous iatrogenic hyponatremia associated, e.g., with the use of glucose-containing infusion solutions.

## Implementation in clinical medicine

Measurement of freezing point depression is the gold standard for determining osmolality. Unfortunately, this method is not carried out in all clinical laboratories, and the results are hardly ever available within a short time. In contrast, blood gas analyzers are normally available near the point of care, and their results can thus be obtained within minutes. Sodium is the primary electrolyte upon which the calculation of osmolality is based, and hypoosmolality is almost always caused by low sodium concentrations. Therefore, the term hyponatremia is very commonly used as a synonym for hypoosmolality in clinical medicine. Importantly, blood gas analyzers can be programmed to precisely calculate osmolality, and the results should be integrated in routine print outs (see above) [14]. In general, clinical attention should be focused on stable osmolality and/or sodium concentrations, and – for the prevention of hyponatremia – it is recommended to use isotonic balanced electrolyte solutions and regularly monitor the acid–base-electrolyte status by blood gas analysis [22, 23].

## Osmolality and the brain

When blood osmolality decreases, water shifts into the brain’s glial cells, causing them to swell (cerebral edema). This results in an increase in brain volume within the rigid skull, which can initially be compensated by a shift of blood and cerebrospinal fluid (CSF) outside of the skull [24]. For example, a decrease in plasma osmolality from 288 to 280 mosmol/kg H_2_O (minus 3%) results in a brain volume increase of 3%, which may cause a decrease in intracranial blood or CSF volume by as much as 30%: normal brain volume 1,340 mL plus 40 mL (3%); normal intracranial blood volume 120 mL minus 40 mL (33%); normal intracranial CSF volume 140 mL minus 40 mL (29%). Upon exceeding a critical limit, intracranial pressure rises substantially and leads to a decrease in cerebral blood flow, possibly resulting in brain herniation, permanent brain damage or death [25, 26].

The risks of hypoosmolality (or hyponatremia) and encephalopathy are higher in hospitalized patients with critical illness or major surgery because stress-induced release of antidiuretic hormone (ADH) results in water retention. Children before puberty and women in the reproductive age group are more vulnerable because of a discrepancy between skull and brain size or a hormonal inhibition of the sodium potassium pumps of the cells, respectively. [24] Hypoosmolality can be caused or aggravated by the use of hypotonic intravenous fluids, e.g., lactated Ringer’s solution (osmolality 256 instead of 288 mosmol/kg H_2_O) or 5% glucose solution (in vivo osmolality 0 instead of 288 mosmol/kg H_2_O; Table 1) [27].

For prevention, the Pharmacovigilance Risk Assessment Committee (PRAC) of the European Medicines Agency (EMA) has recommended that warnings and precautions should be included in the Summaries of Product Characteristics of hypotonic electrolyte solutions [28]. In general, isotonic balanced electrolyte solutions (osmolality 280 to 300 mosmol/kg H_2_O; Table 1) should be preferred in everyday routine for safety reasons, especially in cases with neurosurgery or craniocerebral trauma [22, 23].

## Osmolality and water intoxication

The total concentration of osmotically active particles (osmoles) in the extracellular fluid is determined by the amount of osmoles divided by the volume of the extracellular fluid. The total body water (TBW) is controlled within narrow ranges by the human body through fluid intake and/or thirst as well as renal water excretion [29]. As a consequence, increased water intake is rarely associated with significant hypoosmolality [30]. Water intoxication can occur if the oral intake of solute-free fluids exceeds the kidneys’ capacity to excrete water, resulting in hypoosmolal hyponatremia. Accordingly, in a systematic review of cases with excessive water intake and hyponatremia, Rangan et al. found a median daily water consumption of 8 L and a median sodium concentration of 118 mmol/L at presentation [31]. Frequent motivators for the increased water consumption were psychogenic, iatrogenic, exercise and habitual polydipsia. The clinical features at presentation were severe in 53% (seizures, coma), moderate in 35% (confusion, vomiting, agitation) and mild in 5% (dizziness, lethargy, cognitive deficit) of cases. Treatment-related complications included osmotic demyelination (3%), rhabdomyolysis (7%) and death (13%). Exercise-associated hyponatremia has also been reported in marathon runners [32].

## Osmolality and mortality

Hypoosmolality and/or hyponatremia (< 135 mmol/L) affect approximately 5% of adults and 35% of hospitalized patients. Even mild hyponatremia is associated with longer hospitalization and increased mortality (Table 3) [33–35]. Experimental studies showed that even asymptomatic animals with severe hyponatremia (< 125 mmol/L) had subclinical brain edema which may cause permanent brain damage [36]. In general, the brain cell volume regulation in hyponatremia is influenced by multiple factors, e.g., sex, age, hormones and hypoxia. Patients most susceptible to death or permanent brain damage are prepubescent children and menstruant women (see above) [24]. Hyponatremia treatment guidelines recommend limiting the correction of severe hyponatremia during the first 24 h to prevent osmotic demyelination syndrome (ODS) [37], but a recent systematic review and meta-analysis including 16 cohort studies with a total of 11,811 patients suggests that slower rates of correction (< 8 mmol/L per 24 h) are associated with an increased risk of mortality compared to rapid correction [38].

**Table 3:** Association between hyponatremia and hospital mortality in a retrospective study by Funk et al. [34]

Sodium (mmol/L)	Hospital mortality (%)	Number of patients
140	15	2025
132	29	370
128	32	72
118	33	32

## Conclusion

The use of osmolality (mosmol/kg H_2_O) instead of osmolarity (mosmol/L) is preferable for clinicians to avoid confusing results when comparing solutions with different water contents. Osmolality (normal plasma value 288 mosmol/kg H_2_O) can be calculated based on blood gas analysis to facilitate a precise and rapid diagnosis in everyday routine. To avoid unwanted water shifts, intravenous fluids for replacement of extracellular fluid or plasma should be isotonic with a calculated actual (in vivo) osmolality of 280 to 300 mosmol/kg H_2_O. Hypoosmolality is almost always caused by low sodium concentrations, occurs frequently in hospitalized patients and is associated with significant morbidity and mortality. Recent evidence suggests that slower rates of correction are associated with increased mortality compared to rapid correction.

## Miscellaneous

1. In a case report, coconut water was suggested as a short-term intravenous hydration fluid, but it is not an ideal resuscitation fluid because of its low in vivo osmolality (without glucose) and its low sodium, high potassium and high glucose concentrations [39].
2. The determination of osmolality can be used as a sensitive tool for the monitoring of alcohol (ETOH) intoxication. A blood ETOH concentration of 1‰ causes an osmolar gap of 32.0 mosmol/kg H_2_O in plasma; the difference between the measured and calculated osmolality is about 32.0 mosmol/kg H_2_O [40].

## Supplementary Information

Additional file 1.

## Data Availability

No datasets were generated or analysed during the current study.
