# Supplementary material for: Osmolality (mosmol/kg H2O) versus osmolarity (mosmol/L): applied physiology to improve patient safety
Source: Eur J Med Res. 2025 Dec 7;30:1227. doi: 10.1186/s40001-025-03652-7 (PMC12690852; doi:10.1186/s40001-025-03652-7)
Supplement: Supplementary file 1 — Additional file 1. [file 40001_2025_3652_MOESM1_ESM.docx]

Appendix

**Examples of publications with wrong or inconsistent units for or definitions of osmolarity, osmolality or tonicity**

*- in alphabetical order according to first author’s name -*

Arabi YM, Belley-Cote E, Carsetti A et al.

European Society of Intensive Care Medicine clinical practice guideline on fluid therapy in adult critically ill patients. Part 1: the choice of resuscitation fluids.

Intensive Care Med 2024; 50: 813-831

- Generally, crystalloids rather than albumin are recommended by the authors for volume expansion in most critically ill adult patients, and balanced crystalloids rather than isotonic saline are suggested for volume expansion in this patient population. However, there is no mention of the crucial importance of osmolality for volume replacement.
- Only for traumatic brain injury is it suggested to use isotonic saline rather than balanced crystalloids; furthermore, it is stated that most of the evidence is based on data from RCTs that used balanced crystalloids with near-normal osmolarity. However, it is more important that osmolality is near-normal, and there are in fact many studies that have compared isotonic normal saline to hypotonic (hypoosmolal) balanced solutions.

Balzer C, Cleveland WJ, Li Z, Riess ML

Buffer glucose adjustment affects myocardial function after ischemia–reperfusion in long-term diabetic rat isolated hearts

Physiological Reports 2022; 10: e15387

- As stated by the authors, osmolarity was calculated based on a formula described by Fazekas et al. (Intensive Care Med 2012; DOI 10.1007/s00134-012-2691-0).
- However, this formula was developed to calculate osmolality, not osmolarity (as claimed by the authors).

Bampoe S, Odor PM, Dushianthan A et al.

Perioperative administration of buffered versus non-buffered crystalloid intravenous fluid to improve outcomes following adult surgical procedures.

Cochrane Database of Systematic Reviews 2017, Issue 9. Art. No.: CD004089.

- The authors state that they only included studies that used isotonic fluids (osmolarity 250 to 350 mosmol/L).
- However, osmolarity is not the relevant parameter for isotonicity (instead, osmolality is more appropriate).
- Furthermore, 250 to 350 mosmol/L is a very broad range to define isotonic solutions and denotes solutions as “isotonic” that are clearly hypotonic; rather, 280 to 300 mosmol/kg H_2_O would be more appropriate.

Benzon HA, Bobrowski A, Santhanam Suresh S et al.

Impact of preoperative hyponatraemia on paediatric perioperative mortality

Br J Anaesth 2019; 123: 618-626

- Although the main problem of hyponatraemia and its impact on mortality is the resulting hypoosmolality, the term “osmolality” is not mentioned at all.
- The term “osmolarity” is only mentioned once, without any data and only in the context of urine composition (“urine osmolarity”), without any data or explanation.
- Tonicity is only mentioned in connection with intravenous fluids (hypotonic and isotonic fluids), but there is no explanation or definition of this term.

Berska J, Bugajska J, Sztefko K

The accuracy of serum osmolarity calculation in small children

J Med Biochem 2022; 41: 1-11

- Although the title explicitly refers to osmolarity, the very first sentence of the abstract already refers to measured or calculated osmolality.
- In the further course of the text, the terms “osmolality” and “osmolarity” are patently used somewhat randomly and synonymously.
- For example, in Table 1, osmolality is claimed to be calculated as mosmol/L, but in Table 3 the units for calculated osmolality are correctly given as mosmol/kg H_2_O.
- Confusion between calculated osmolarity, calculated osmolality and measured osmolality is obvious.

Blank SP, Blank RM

Mind the gap: Measured and calculated osmolarity are not interchangeable in diabetic hyperglycemic emergencies

Exp Clin Endocrinol Diabetes 2023; 131: 268-273

- There is a mix-up of the terms “osmolarity” and “osmolality”. In fact, in diabetic hyperglycaemic emergencies, osmolality (and not osmolarity) is the most important cause of organ damage.
- Osmolarity is claimed to have been calculated using the formula “2Na + glucose + urea (all values in mmol/L)”. Besides the fact that this is the wrong unit for osmolarity (*recte*: mosmol/L), this formula has been developed to calculate actual osmolality (mosmol/kg H_2_O); see Fazekas et al. Intensive Care Med 2012; DOI 10.1007/s00134-012-2691-0.

Boer C, Bossers SM, Koning NJ

Choice of fluid type: physiological concepts and perioperative indications

Br J Anaesth 2018; 120: 384-396

- A table gives an overview of crystalloid and colloid fluids commonly used in the perioperative setting, stating for example the osmolarity of normal saline (308 mosmol/L) as well as dextrose 5% (278 mosmol/L).
- There is no mention of the clinically relevant fact that the stated osmolarity of dextrose 5% is only the *in vitro* osmolarity, whereas the *in vivo* osmolarity of dextrose 5% is 0 mosmol/L.
- There is no mention of osmolality.

Brossier DW, Tume LN, Briant AR et al. for the Metabolism Endocrinology and Nutrition section of the European Society of Pediatric and Neonatal Intensive Care (ESPNIC)

ESPNIC clinical practice guidelines: intravenous maintenance fluid therapy in acute and critically ill children- a systematic review and meta-analysis

Intensive Care Med 2022; 48: 1691-1708

- One of the 5 key questions in this guideline is as follows: “Do isotonic solutions versus hypotonic solutions impact on clinical outcomes?” However, there is no explanation or definition of isotonic or hypotonic fluids.
- There is no mention of osmolality – the key parameter for the tonicity of IV fluids –in the whole article.
- The term osmolarity is only mentioned once (in the context of compatibility with peripheral infusion), but there is no mention of the unit.

Deißler L, Wirth R, Frilling B, Janneck M, Rösler A:

Hydration status assessment in older patients.

Dtsch Arztebl Int 2023; 120: 663-669

- Inconsistent and wrong units for osmolality in Tables 1-3: mmol/L, mosmol/L or mosmol/kg (*recte*: mosmol/kg H_2_O).
- Hyperosmolal dehydration is defined as a serum osmolality ≥ 295 mmol/L (*recte*: mosmol/kg H_2_O).

Feld LG, Neuspiel DR, Foster BA, et al.

Clinical Practice Guideline: Maintenance Intravenous Fluids in Children

Pediatrics 2018; 142: e20183083

- A table showing the composition of commonly used maintenance fluids and human plasma states the (theoretical) plasma osmolarity to be 308 mosmol/L (*recte*: 291 mosmol/L).
- In the same table, the plasma osmolality is given as 275-295 mosmol/kg (*recte*: 287 ± 5 mosmol/kgH_2_O)

Hahn RG

Should anaesthetists stop infusing isotonic saline? (editorial)

Br J Anaesth 2014; 112: 4-6

- Data for osmolality are presented with wrong units and wrong numbers, e.g.:
- Plasma 295-300  mosmol/kg (*recte*: 287 ± 5 mosmol/kgH_2_O)
- Isotonic saline 308 mosmol/kg (*recte*: 286 mosmol/kgH_2_O)

Hayes W.

Ab-normal saline in abnormal kidney function: risks and alternatives.

Pediatr Nephrol 2019; 34: 1191-1199

- Wrong definition and unit for osmolality in Table 1: osmolality [mosmol/L] (*recte*: mosmol/kg H_2_O); likely confused with osmolarity.
- According to Table 1, plasma has an osmolality of 275-295 mosmol/L (*recte*: 286-290 mosmol/kg H_2_O), NaCl 0,9% has an osmolality of 308 mosmol/L (*recte*: 286 mosmol/kg H_2_O), and Plasmalyte 148 has an osmolality of 295 mosmol/L (*recte*: 271 mosmol/kg H_2_O).
- Consequently, 0.9% saline is falsely stated to be a hypertonic solution: “For children with traumatic brain injury, the hypertonicity of 0.9% saline may mitigate the risk of cerebral edema more effectively than balanced isotonic fluids […]”. However, normal saline is in fact nearly isotonic, whereas Plasmalyte 148, often compared to normal saline, is clearly hypotonic rather than isotonic.
- As a consequence, the supposedly correct answer to question 2 of the closing multiple-choice questions (Q: “0.9% saline has the following properties, relative to plasma”; A: “tonicity 110% Na-concentration 110%, Cl-concentration 160%”) is not correct, because normal saline is not hypertonic but rather isotonic (“tonicity 100%”).

Hoorn EJ

Intravenous fluids: balancing solutions.

J Nephrol 2017; 30: 485-492.

- The values presented as the osmolality of plasma and intravenous fluids are given in mosmol/kg (*recte*: mosmol/kg H_2_O).

MacDonald N, Pearse R M

Are we close to the ideal intravenous fluid?

Br J Anaesth 2017; 119 (suppl1): i63-i71

- There are several inaccurate statements, e.g.:
- Ringer´s lactate and Plasmalyte are claimed to be isotonic (*recte*: hypotonic).
- Normal saline is claimed to be hypertonic (*recte*: isotonic).
- Although tonicity is one of the central topics of this article, there is no mention of osmolality (not even of osmolarity).

Malbrain ML, Wong A, Nasa P, Ghosh S (eds)

Rational Use of Intravenous Fluids in Critically Ill Patients.

2024 Springer Heidelberg New York

- Osmolality, osmolarity and tonicity are key terms in this book. However, there are inconsistent and contradictory definitions and wrong units used in connection with these terms in various chapters of the book.
- Definition of osmolality (p. 25): “A measure of the concentration of osmotically active particles per unit volume of solution, measured in milliosmoles per litre of solution (mosmol/l).” In fact, the authors inaccurately claim: “In clinical practice, osmolarity and osmolality are similar enough to be used interchangeably”.
- Definition of osmolarity (p. 25): “A measure of the concentration of osmotically active particles per unit mass of solution, measured in milliosmoles per kilogram of solution (mosm/kg)”.
- The definition of osmolarity in this chapter patently is the correct definition of osmolarity, and *vice versa*. However, the correct unit for osmolality is mosmol/kg H_2_O (and not mosmol/kg).
- Definition of tonicity (p. 38): “The tonicity determines the distribution of the given solution. The cell will shrink when placed in a hypertonic solution, swell when placed in a hypotonic solution and the cell volume will remain unchanged when placed in an isotonic solution.” In the further course of the text (chapter 9, p. 211), it is claimed that “normal plasma tonicity is 270–290 mOsm/kg”; however, tonicity is a dimensionless measure.

McLean DJ, Shaw AD

Intravenous fluids: effects on renal outcomes

Br J Anaesth 2018; 120: 397e402

- It is certainly true, that – as the authors state – “the kidney is a dynamic organ that plays a fundamental role in regulating plasma osmolality […]”; however, there is no further explanation of the term osmolality, and there are no units given.
- The term osmolarity is not mentioned at all.
- According to the authors, the most important distinction for crystalloids is between balanced and unbalanced chloride-rich fluids. However, the distinction between hypotonic and isotonic solutions is at least as important. Furthermore, the balanced solutions mentioned by the authors (Ringer’s solution and Plasma-Lyte) are both clearly hypotonic.

Pfortmueller CA, Funk G-C, Reiterer C et al.

Normal saline versus a balanced crystalloid for goal-directed perioperative fluid therapy in major abdominal surgery: a double-blind randomised controlled study

Br J Anaesth 2018; 120: 274-283

- The theoretical osmolality of normal saline is claimed to be 308 mosmol/kg.
- However, this is the wrong term, unit or number. For theoretical osmolarity, the correct unit is mosmol/L; for theoretical osmolality, it is mosmol/kg H_2_O (with nearly equal numerical values for normal saline), but more important is the actual (real) osmolality, which for normal saline is 286 mosmol/kg H_2_O (see also the comment below on Potura et al. 2015).

Potura E, Lindner G, Biesenbach P et al.:

An acetate-buffered balanced crystalloid versus 0.9% saline in patients with end-stage renal disease undergoing cadaveric renal transplantation: a prospective randomized controlled trial.

Anesth Analg 2015; 120: 123 – 129

- The authors claim that normal saline has a theoretical osmolality of 308 mosmol/kg and that Elomel-Isoton has a theoretical osmolality of 302 mosmol/kg.
- These are wrong terms, units or numbers. For theoretical osmolarity, the correct unit is mosmol/L, and for theoretical osmolality, it is mosmol/kg H_2_O. The numerical values of theoretical osmolarity and theoretical osmolality are virtually identical because the water content of a crystalloid solution is very close to 1 (0.997); but more important is the actual (real) osmolality (= theoretical osmolality times osmotic coefficient), which for normal saline results in 286 mosmol/kg H_2_O and for Elomel-Isoton is 280 mosmol/kg H_2_O.

Rasouli M

Basic concepts and practical equations on osmolality: Biochemical approach

Clinical Biochemistry 2016; 49: 936 – 941

- There is a mix-up between the units for osmolarity and osmolality: although the definition for osmolality is correctly given as the numbers of milliosmoles of solutes per one kilogram of water (mosmol/kg H_2_O) and the definition for osmolarity as the numbers of milliosmoles of solutes per liter of solution (mosmol/L), there is also the statement that the typical plasma osmolality is 285 mosmol/L.
- In the abstract it is stated that the osmolality of plasma is calculated by dividing the osmolarity by the plasma water. The author also claims that the water content of plasma is normally about 0.93 kg H_2_O/L plasma. This would result in an abnormal plasma osmolality of 313 mosmol/kg H_2_O (calculation: 291 (plasma osmolarity)/0.93 (water content) = 313). In contrast, the normal osmolality of plasma measured by freezing point depression is 288 mosmol/kg H_2_O, and the normal osmolality of plasma calculated with adequate formulas is 287 mosmol/kg H_2_O (Fazekas et al. Intensive Care Med 2012; DOI 10.1007/s00134-012-2691-0).

Van Regenmortel N, De Weerdt T, Van Craenenbroeck AH, et al.

Effect of isotonic vs hypotonic maintenance fluid therapy on urine output, fluid balance, and electrolyte homeostasis: a crossover study in fasting adult volunteers.

Br J Anaesthesia 2017; 118: 892–900

- In this article, tonicity and osmolality are central topics, but there are no definitions of the two terms, and the units used are wrong.
- An IV fluid consisting of NaCl 0.9% in glucose 5% with an added 40 mmol of potassium chloride 7.45% is claimed to have an osmolarity of 614 mosmol/L and a tonicity of 373 mosmol/L; however, tonicity is a dimensionless measure describing whether a cell exposed to osmotic pressure will swell, shrink, or remain the same size.
- In Figure 2, data for urine osmolality are presented, but the y-axis is labelled inaccurately, namely urine sodium [mmol/l] instead of osmolality [mosmol/kg H_2_O].
- In Figure 3, data for serum osmolality are given as mosmol/kg (*recte*: mosmol/kg H_2_O).

Sakka SG

Kalium – was Intensivmedizinerinnen und Intensivmediziner wissen sollten (Potassium – what an intensive care specialist should know; article in German)

Anästh Intensivmed 2023; 64: 447-459

- Osmolality and osmolarity are patently used interchangeably.
- Reference is made to the influence of osmolality on serum-potassium levels, but it is recommended to measure osmolarity.

Vujovic P, Chirillo M, Silverthorn DU

Learning (by) osmosis: an approach to teaching osmolarity and tonicity

Adv Physiol Educ 2018; 42: 626 – 635

- It is correct that “osmolarity is a measure of the concentration of osmotically active particles in a solution, and the relevant unit is mosmol/L.
- However, the physiological effect is related to the concentration of osmotically active particles within the water space of such a solution; therefore, the unit is mosmol/kg H_2_O.
- As a result, the following statement is wrong: ”You can measure the osmolarity of a solution using a machine called an osmometer. The most common commercial osmometers measure either freezing point depression or vapor pressure of a sample of solution.”
- The correct statement is: *Common commercial osmometers measure osmolality in mosmol/kg H_2_O*.
